# Supplementary material for: Nature and determinants of social actions during a mass shooting
Source: PLoS One. 2021 Dec 7;16(12):e0260392. doi: 10.1371/journal.pone.0260392 (PMC8651140; doi:10.1371/journal.pone.0260392)
Supplement: S2 File — (DOC) [file pone.0260392.s002.doc]

# Questionnaire “Bataclan”

# Instructions générales pour mener l’entretien

NB : Ce questionnaire ne peut être proposé au participant qu’après la signature du consentement. L’expérimentateur (vous-même) rappelle au participant qu’il est libre d’interrompre l’entretien à tout moment sans avoir à apporter de justification. Merci également de demander au participant s’il accepte que sa voix soit enregistrée (elle ne sera utilisée que pour enrichir les réponses au questionnaire, aucune utilisation publique de la voix ne sera faite).

- Le questionnaire comprend différentes étapes : premièrement, des informations démographiques ; deuxièmement, des informations sur la situation du participant le soir du 13/11/2015 ; troisièmement, ses perceptions et comportements lorsque qu’il s’est rendu compte que quelque chose de grave se passait mais qu’il ne savait pas encore de quoi il s’agissait ; quatrièmement, ses perceptions et comportements lorsqu’il s’est rendu compte qu’il s’agissait d’une attaque à l’arme à feu ; cinquièmement, ses perceptions et comportements jusqu’à la sortie du Bataclan ; enfin et sixièmement, des questions annexes et des commentaires libres.
- Les questions seront à dicter au participant (rester le plus fidèle possible aux MOTS employés dans le questionnaire). Vous coterez vous-mêmes ses réponses. Le participant commente librement ses réponses, sur le ou les point-s qu’il souhaite illustrer, exemplifier ou détailler. Le micro reste ouvert pendant toute la durée de l’entretien.
- Merci de demander au participant de répondre le plus sincèrement possible à toutes les questions en essayant de se rappeler au maximum des pensées, croyances et émotions qu’il/elle ressentait au moment précis.
- Si parmi les réponses, aucune ne convient exactement au participant, demandez celle qu’il/elle pense être la plus proche de sa situation personnelle.
- A la fin de l’entretien, demandez au participant de ne pas communiquer jusqu’à la fin de l’étude à propos du contenu de cet entretien aux autres personnes présentes le soir du 11/13/2015 au Bataclan, et avec qui il serait susceptible d’interagir par la suite.

**CHECK DE LA PROCEDURE**

Notice d’information adulte lue et connue

Consentements signés (3 copies paraphées à chaque page)

Micro ouvert

Lecture des instructions au participant

# **Date et heure de début d’entretien : ________________**

# **Initiales de l’expérimentateur : ________________**

#

# Partie A. Informations démographiques

« Cet entretien se fera en 6 parties. Dans cette première partie, je vais vous demander des informations d’ordre général. »

**A1. Votre sexe** (cocher une seule case)

Homme  Femme

**A2. Votre mois et année de naissance**

Mois  Année

**A3. Votre niveau d’études** (cocher une seule case)

Avant le BEP/CAP

Bac

Bac+3

Bac +5

Plus de Bac +5

**A4. Votre catégorie socio-professionnelle** (cocher une seule case)

Agriculteurs exploitants

Artisans, commerçants et chefs d’entreprise

Cadres et professions intellectuelles supérieures

Professions intermédiaires

Employés

Ouvriers

**A5. Votre statut matrimonial** (cocher une seule case)

Marié

Divorcé/séparé

En concubinage

Célibataire

**A6. Avez-vous des enfants ?** (cocher une seule case)

Oui - combien : **_________________ ?**

Non

# Partie B. Informations contextuelles

« Dans cette deuxième partie de l’entretien, je vais vous poser des questions sur le soir du 13 novembre 2015. »

**B1. Etiez-vous en présence d’un ou de plusieurs membres de votre famille au Bataclan le soir du 13 novembre ?**

Oui y

Non

Si oui

Père

Mère

Frère(s) et combien : **_________________ ?**

Sœur(s) et combien : **_________________ ?**

Fils et combien : **_________________ ?**

Fille(s) et combien : **_________________ ?**

**B2. Etiez-vous en présence de votre conjoint au Bataclan le soir du 13 novembre ?** (cocher une seule case)

Oui

Non

Non-applicable (célibataire)

**B3. Etiez-vous en présence d’amis au Bataclan le soir du 13 novembre ?** (cocher une seule case)

Oui - et combien : **_________________ ?**

Non

**B4. Avez-vous été touché par un projectile (balle) ?** (cocher une seule case)

Oui

Non

**B5. Avez-vous été opéré à la suite des événements ?** (cocher une seule case)

Oui

Non

# Partie C. « Quelque chose de grave se passait mais je ne savais pas quoi. »

« Je vais à présent vous poser quelques questions concernant le tout début de l’attaque, **lorsque vous saviez que quelque chose de grave se passait mais que vous ne saviez pas quoi exactement.** Pour répondre à ces questions, il est important de **vous concentrer sur les pensées, émotions et croyances que vous avez eues à ce moment précis.**»

**C1.** « Je vais d’abord vous demander d’annoter ce plan du Bataclan de la manière suivante : **une croix verte pour où vous étiez à ce moment-là**; **une croix bleue pour l’endroit où vous pensiez que chacune de vos connaissances était à ce moment précis (famille, conjoint, amis – une croix par connaissance)**; **une croix rouge pour l’endroit où vous pensiez que la menace non-identifiée se trouvait - une croix par location envisagée**. ». Merci de répondre en essayant de vous rappeler de vos souvenirs à ce moment-là : si vous pensiez que la menace venait de deux endroits différents, merci de mettre deux croix rouges, etc. »


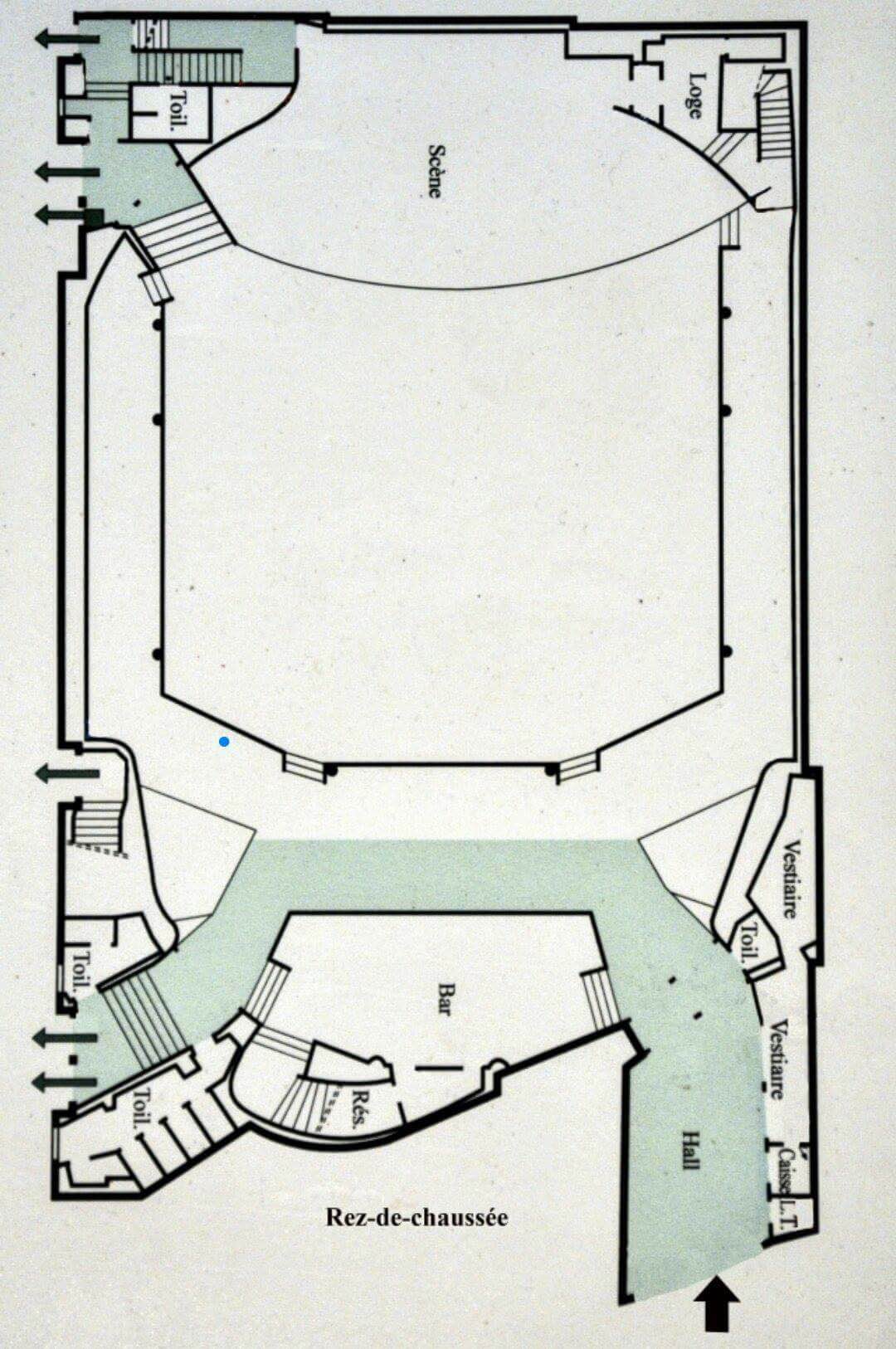


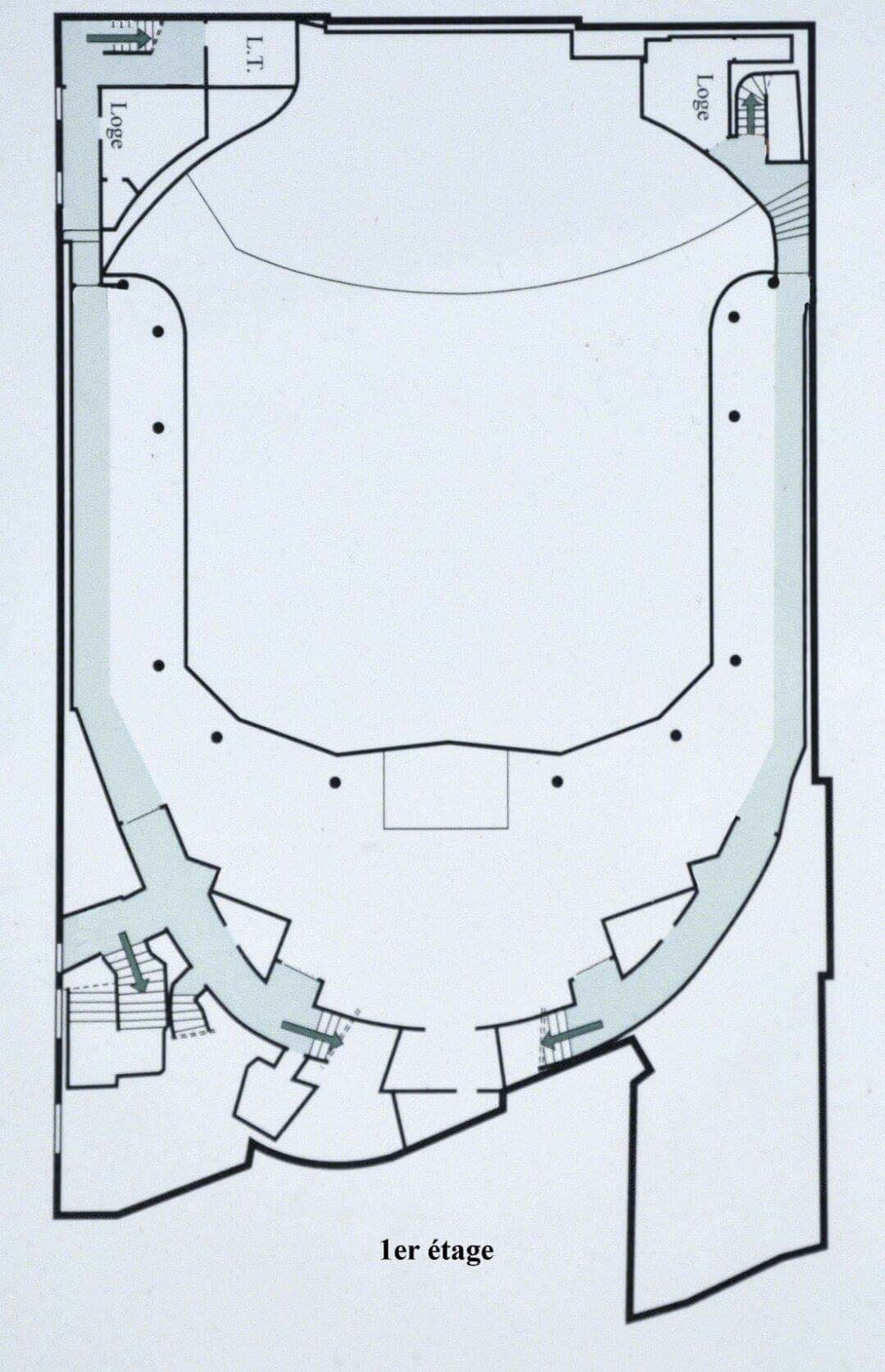


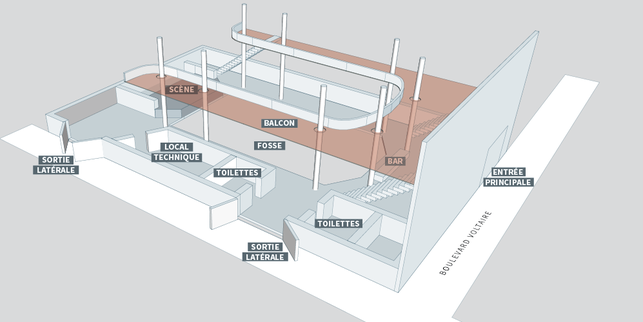


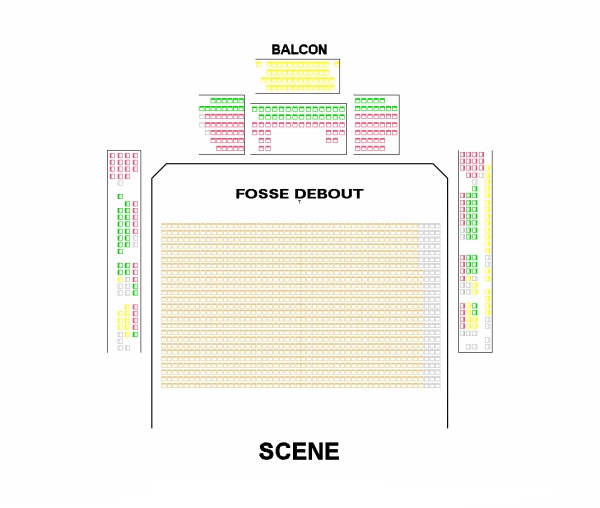


**C2.** « Je vais vous poser quelques questions sur vos émotions et pensées à ce moment-précis ».

**C2.1 Aviez-vous l’impression que votre vie pouvait être en danger ?** (cocher une seule case)

Oui

Non

Je ne sais pas

**C2.2 Aviez-vous l’impression que la vie de vos connaissances** (si applicable) **pouvait être en danger ?** (cocher une seule case)

Oui

Non

Je ne sais pas

Non-applicable (seul dans la salle)

**C2.3 L’évacuation vous semblait-elle possible ?** (cocher une seule case)

Oui

Non

**C3.** « Je vais à présent vous lire des propositions. Pourriez-vous m’indiquer si elles correspondent ou non à ce que vous avez fait ou observé **à ce moment précis** (**lorsque vous saviez que quelque chose de grave se passait mais que vous ne saviez pas quoi exactement**). Avez-vous vu ou fait ces choses : pas du tout, au moins une fois, plus d’une fois, ou un grand nombre de fois.»

|  | **Pas du tout** | **Au moins une fois** | **Plus d’une fois** | **Un grand nombre de fois** |
| --- | --- | --- | --- | --- |
| **Réactions spontanées** | **0** | **1** | **2** | **3** |
| C3.1.a Avez-vous couru ou cherché à vous enfuir ? |  |  |  |  |
| C3.1.b Avez-vous vu des personnes courir ou chercher à s’enfuir ? |  |  |  |  |
| C3.2.a Avez-vous hurlé ? |  |  |  |  |
| C3.2.b Avez-vous entendu des personnes hurler ? |  |  |  |  |
| C3.3.a Vous-êtes-vous recroquevillé ? |  |  |  |  |
| C3.3.b Avez-vous vu des personnes se recroqueviller ? |  |  |  |  |
| C3.4.a Avez-vous bousculé une personne pour vous protéger ? |  |  |  |  |
| C3.4.b Avez-vous vu des personnes en bousculer d’autres pour se protéger ? |  |  |  |  |
| C3.5.a Êtes-vous resté immobile ? |  |  |  |  |
| C3.5.b Avez-vous vu des personnes rester immobile ? |  |  |  |  |
| C3.6.a Avez-vous essayé d’en savoir plus sur la menace ? |  |  |  |  |
| C3.6.b Avez-vous eu l’impression que les personnes autour de vous essayaient d’en savoir plus sur la menace ? |  |  |  |  |
| C3.7.a Avez-vous cherché à vous agripper à quelqu’un ? |  |  |  |  |
| C3.7.b Les gens cherchaient-ils à s’agripper à une autre personne ? |  |  |  |  |
| **Relations à l’autre** | **0** | **1** | **2** | **3** |
| C3.7.a Avez-vous protégé une personne de votre corps ? |  |  |  |  |
| C3.7.b Avez-vous vu des personnes en protéger d’autres par leur corps ? |  |  |  |  |
| C3.8.a Vous-êtes-vous mutuellement protégé avec quelqu’un ? |  |  |  |  |
| C3.8.b Avez-vous des personnes se protéger mutuellement par leur corps ? |  |  |  |  |
| C3.9.a Avez-vous tenté de rassurer quelqu’un (texto, geste, parole) ? |  |  |  |  |
| C3.9.b Avez-vous vu des personnes tenter d’en rassurer d’autres (texto, geste, parole) ? |  |  |  |  |
| C3.10.a Avez-vous appelé à l’aide (texto, geste, parole) ? |  |  |  |  |
| C3.10.b Avez-vous vu des personnes en appeler d’autres à l’aide (texto, geste, parole) ? |  |  |  |  |
| C3.11.a Avez-vous apporté de l’aide à quelqu’un ? |  |  |  |  |
| C3.11.b Avez-vous vu des personnes apporter de l’aide à une autre ? |  |  |  |  |
| **Communication avec les autres otages** | **0** | **1** | **2** | **3** |
| C3.12.a Avez-vous informé autrui de la nature de la menace (texto, geste, parole) ? |  |  |  |  |
| C3.12.b Avez-vous vu des personnes informer autrui de la nature de la menace  (texto, geste, parole) ? |  |  |  |  |
| C3.13.a Avez-vous informé autrui de la position de la menace (texto, geste, parole) ? |  |  |  |  |
| C3.13.b Avez-vous vu des personnes informer autrui de la position de la menace (texto, geste, parole) ? |  |  |  |  |
| C3.14.a Avez-vous informé autrui de la position des issues de secours (texto, geste, parole) ? |  |  |  |  |
| C3.14.b Avez-vous vu des personnes informer autrui de la position des issues de secours (texto, geste, parole) ? |  |  |  |  |
| C3.15.a Avez-vous informé autrui de votre blessure (si applicable) (texto, geste, parole) ? |  |  |  |  |
| C3.15.b Avez-vous vu des personnes informer autrui de leur blessure (texto, geste, parole) ? |  |  |  |  |
| **Communication avec l’extérieur** | **0** | **1** | **2** | **3** |
| C3.16 Avez-vous tenté de communiquer avec l’extérieur (texto, appel, social media) ? |  |  |  |  |

# C4. « Le dessin ci-dessous représente votre ‘relation à autrui’. En A, vous vous sentiez totalement indépendant d’autrui. En E, vous sentiez que vous-mêmes et autrui ne faisiez qu’un. Pourriez-vous me dire quelle situation correspondait à votre ressenti d’alors (lorsque vous saviez que quelque chose de grave se passait mais que vous ne saviez pas quoi exactement) envers (une réponse par item) : »

# Vos proches (famille, amis, connaissances) présents au Bataclan _________________ ?

# Vos proches (famille, amis, connaissances) non-présents au Bataclan _________________ ?

# Le reste de la foule _________________ ?

# Le groupe Eagles of Death Metal _________________ ?

#
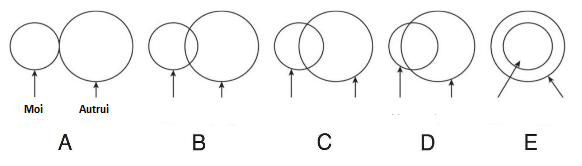


# Partie D. « Je me suis rendu compte qu’il s’agissait d’une attaque à l’arme à feu. »

« Je vais à présent vous poser quelques questions concernant la suite, **lorsque vous vous êtes rendus compte qu’il s’agissait d’une attaque à l’arme à feu.** Pour répondre à ces questions, il est important de garder en tête **qu’il s’agit de vous concentrer sur les pensées, émotions et croyances que vous avez eues à ce moment précis.**»

**D1.** « Je vais d’abord vous demander d’annoter ce plan du Bataclan de la manière suivante : **une croix verte pour où vous étiez à ce moment-là**; **une croix bleue pour l’endroit où vous pensiez que chacune de vos connaissances était à ce moment précis (famille, conjoint, amis – une croix par connaissance)**; **une croix rouge pour l’endroit où vous pensiez que les assaillants étaient à ce moment précis - une croix par assaillant**.  Merci de répondre en essayant de vous rappeler de vos souvenirs à ce moment-là : si vous pensiez qu’ils étaient deux, n’en mettez que 2, etc. »


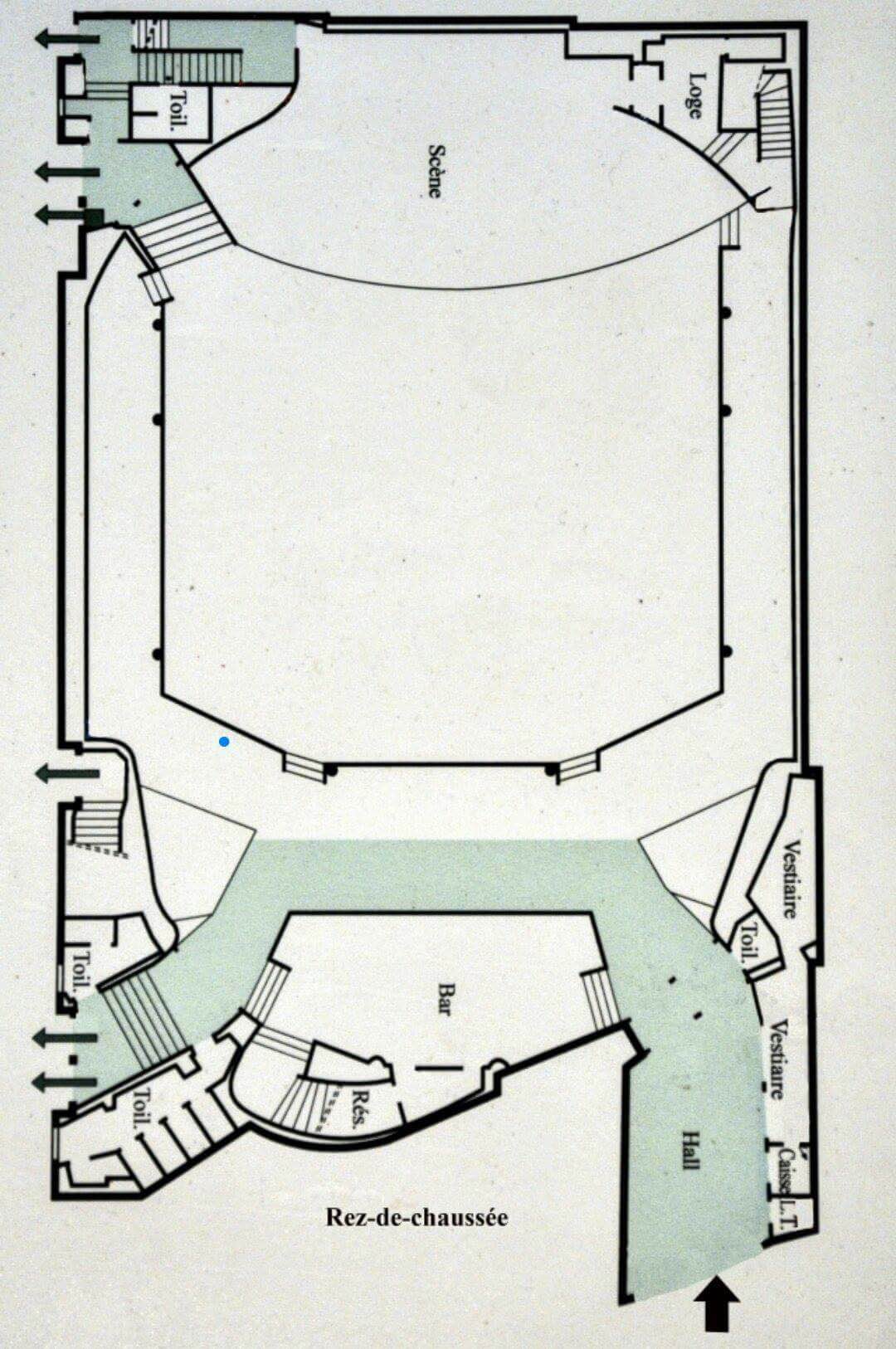


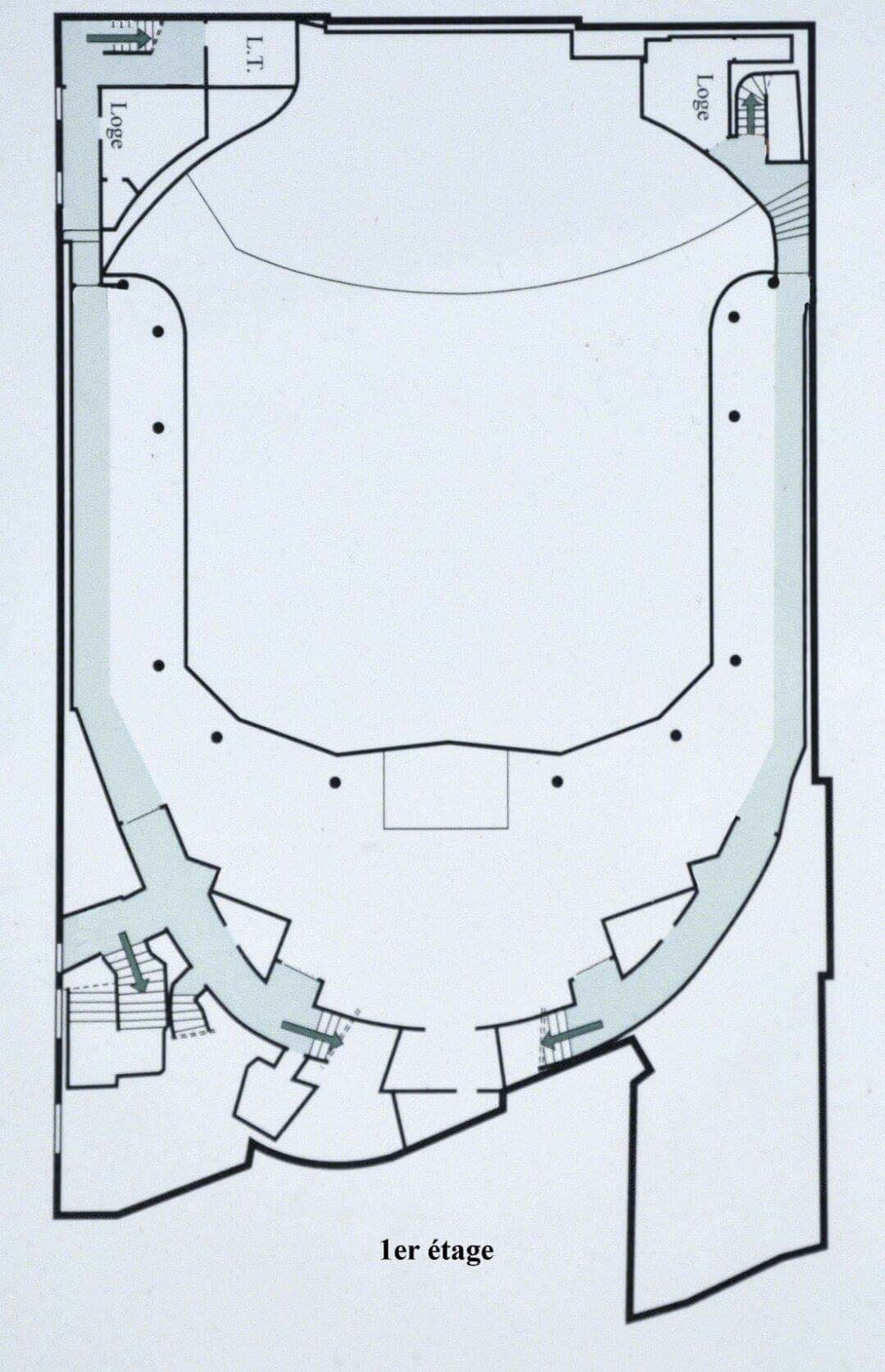


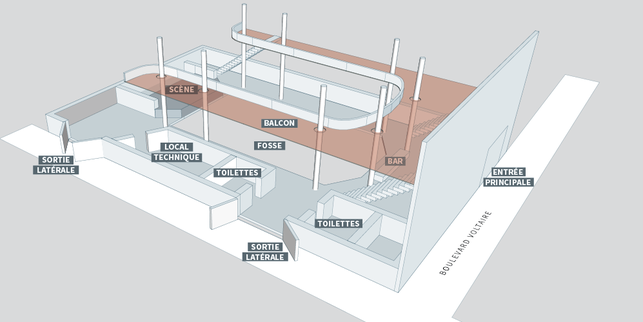


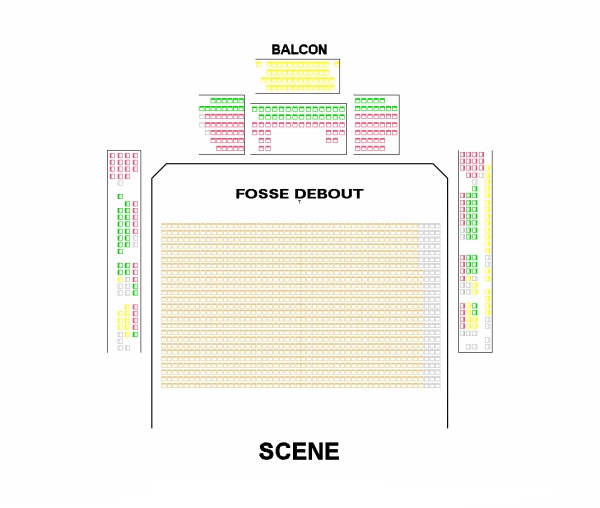


**D2.** « Je vais vous poser quelques questions sur vos émotions et pensées à ce moment-précis ».

**D2.1 Aviez-vous l’impression que votre vie pouvait être en danger ?** (cocher une seule case)

Oui

Non

Je ne sais pas

**D2.2 Aviez-vous l’impression que la vie de vos connaissances** (si applicable) **pouvait être en danger ?** (cocher une seule case)

Oui

Non

Je ne sais pas

Non-applicable (seul dans la salle)

**D2.3 L’évacuation vous semblait-elle possible ?** (cocher une seule case)

Oui

Non

**D3.** « Je vais à présent vous poser quelques questions concernant la suite, **lorsque vous vous êtes rendus compte qu’il s’agissait d’une attaque à l’arme à feu.** Pour répondre à ces questions, il est important de garder en tête **qu’il s’agit de vous concentrer sur les pensées, émotions et croyances que vous avez eues à ce moment précis.** Avez-vous vu ou fait ces choses : pas du tout, au moins une fois, plus d’une fois, ou un grand nombre de fois.»

(cocher une seule case par item)

|  | **Pas du tout** | **Au moins une fois** | **Plus d’une fois** | **Un grand nombre de fois** |
| --- | --- | --- | --- | --- |
| **Réactions spontanées** | **0** | **1** | **2** | **3** |
| D3.1.a Avez-vous couru ou cherché à vous enfuir ? |  |  |  |  |
| D3.1.b Avez-vous vu des personnes courir ou chercher à s’enfuir ? |  |  |  |  |
| D3.2.a Avez-vous hurlé ? |  |  |  |  |
| D3.2.b Avez-vous entendu des personnes hurler ? |  |  |  |  |
| D3.3.a Vous-êtes-vous recroquevillé ? |  |  |  |  |
| D3.3.b Avez-vous vu des personnes se recroqueviller ? |  |  |  |  |
| D3.4.a Avez-vous bousculé une personne pour vous protéger ? |  |  |  |  |
| D3.4.b Avez-vous vu des personnes en bousculer d’autres pour se protéger ? |  |  |  |  |
| D3.5.a Êtes-vous resté immobile ? |  |  |  |  |
| D3.5.b Avez-vous vu des personnes rester immobile ? |  |  |  |  |
| D3.6.a Avez-vous essayé d’en savoir plus sur la menace ? |  |  |  |  |
| D3.6.b Avez-vous eu l’impression que les personnes autour de vous essayaient d’en savoir plus sur la menace ? |  |  |  |  |
| D3.7.a Avez-vous cherché à vous agripper à quelqu’un ? |  |  |  |  |
| D3.7.b Les gens cherchaient-ils à s’agripper à une autre personne ? |  |  |  |  |
| **Relations à l’autre** | **0** | **1** | **2** | **3** |
| D3.7.a Avez-vous protégé une personne de votre corps ? |  |  |  |  |
| D3.7.b Avez-vous vu des personnes en protéger d’autres par leur corps ? |  |  |  |  |
| D3.8.a Vous-êtes-vous mutuellement protégé avec quelqu’un ? |  |  |  |  |
| D3.8.b Avez-vous des personnes se protéger mutuellement par leur corps ? |  |  |  |  |
| D3.9.a Avez-vous tenté de rassurer quelqu’un (texto, geste, parole) ? |  |  |  |  |
| D3.9.b Avez-vous vu des personnes tenter d’en rassurer d’autres (texto, geste, parole) ? |  |  |  |  |
| D3.10.a Avez-vous appelé à l’aide (texto, geste, parole) ? |  |  |  |  |
| D3.10.b Avez-vous vu des personnes en appeler d’autres à l’aide (texto, geste, parole) ? |  |  |  |  |
| D3.11.a Avez-vous apporté de l’aide à quelqu’un ? |  |  |  |  |
| D3.11.b Avez-vous vu des personnes apporter de l’aide à une autre ? |  |  |  |  |
| **Communication avec les autres otages** | **0** | **1** | **2** | **3** |
| D3.12.a Avez-vous informé autrui de la nature de la menace (texto, geste, parole) ? |  |  |  |  |
| D3.12.b Avez-vous vu des personnes informer autrui de la nature de la menace  (texto, geste, parole) ? |  |  |  |  |
| D3.13.a Avez-vous informé autrui de la position de la menace (texto, geste, parole) ? |  |  |  |  |
| D3.13.b Avez-vous vu des personnes informer autrui de la position de la menace (texto, geste, parole) ? |  |  |  |  |
| D3.14.a Avez-vous informé autrui de la position des issues de secours (texto, geste, parole) ? |  |  |  |  |
| D3.14.b Avez-vous vu des personnes informer autrui de la position des issues de secours (texto, geste, parole) ? |  |  |  |  |
| D3.15.a Avez-vous informé autrui de votre blessure (si applicable) (texto, geste, parole) ? |  |  |  |  |
| D3.15.b Avez-vous vu des personnes informer autrui de leur blessure (texto, geste, parole) ? |  |  |  |  |
| **Communication avec l’extérieur** | **0** | **1** | **2** | **3** |
| D3.16 Avez-vous tenté de communiquer avec l’extérieur (texto, appel, social media) ? |  |  |  |  |

# D4. « Le dessin ci-dessous représente votre ‘relation à autrui’. En A, vous vous sentiez totalement indépendant d’autrui. En E, vous sentiez que vous-mêmes et autrui ne faisiez qu’un. Pourriez-vous me dire quelle situation correspondait à votre ressenti d’alors (lorsque vous vous êtes rendus compte qu’il s’agissait d’une attaque à l’arme à feu) envers (une réponse par item) : »

# Vos proches (famille, amis, connaissances) présents au Bataclan _________________ ?

# Vos proches (famille, amis, connaissances) non-présents au Bataclan _________________ ?

# Le reste de la foule _________________ ?

# Le groupe Eagles of Death Metal _________________ ?

#
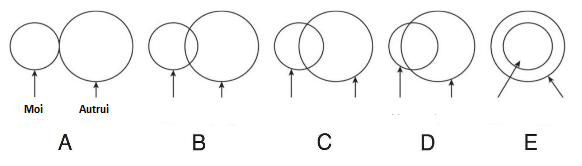


# Partie E. « Jusqu’à la sortie – le temps le plus long. »

« Je vais à présent vous poser quelques questions concernant la suite (le temps le plus long), **jusqu’à votre sortie du Bataclan.** Pour répondre à ces questions, il est important de garder en tête **qu’il s’agit de vous concentrer sur les pensées, émotions et croyances que vous avez eues à ce moment précis. »**

**E1. «**Pourriez-vous m’indiquer si vous êtes sortis (une seule réponse): »

Avant la mort des assaillants

Après la mort des assaillants

**E2.** « Je vais d’abord vous demander d’annoter ce plan du Bataclan de la manière suivante : **une croix verte pour où vous étiez à ce moment-là**; **une croix bleue pour l’endroit où vous pensiez que chacune de vos connaissances était à ce moment précis (famille, conjoint, amis – une croix par connaissance)**; **une croix rouge pour l’endroit où vous pensiez que les assaillants étaient à ce moment précis - une croix par assaillant**. Merci de répondre en essayant de vous rappeler de vos souvenirs à ce moment-là : si vous pensiez qu’ils étaient deux, n’en mettez que 2, etc. »


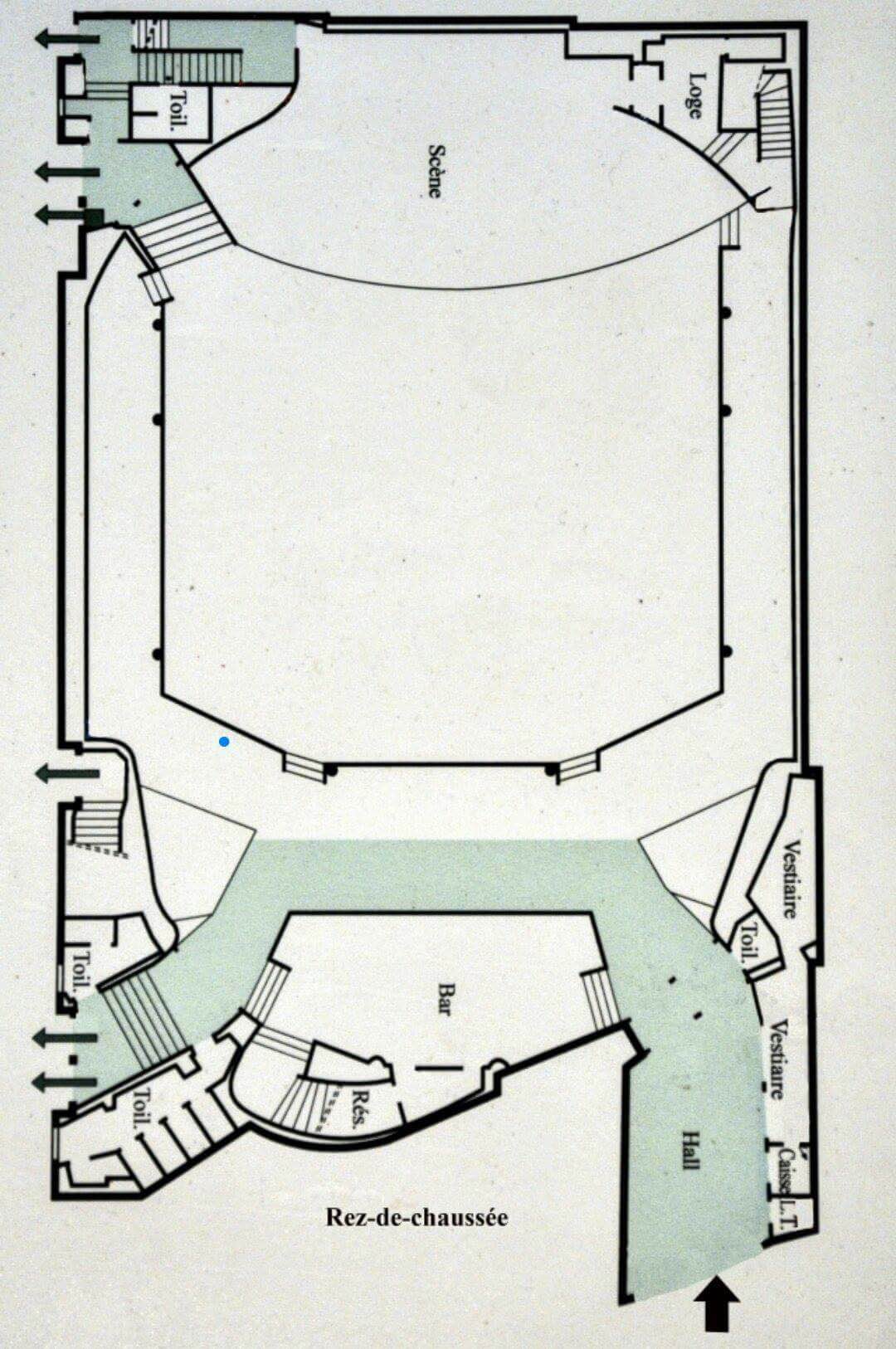

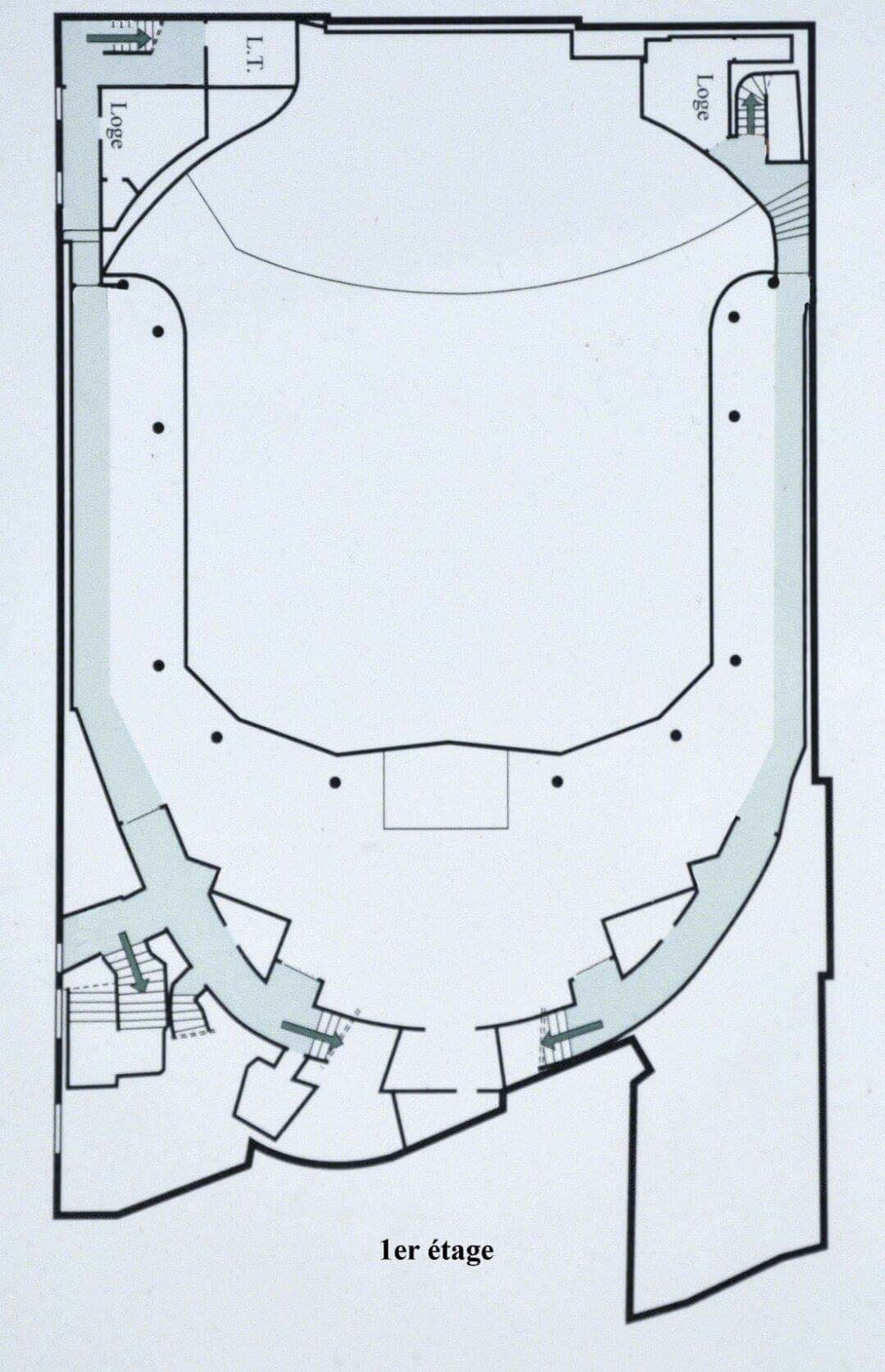


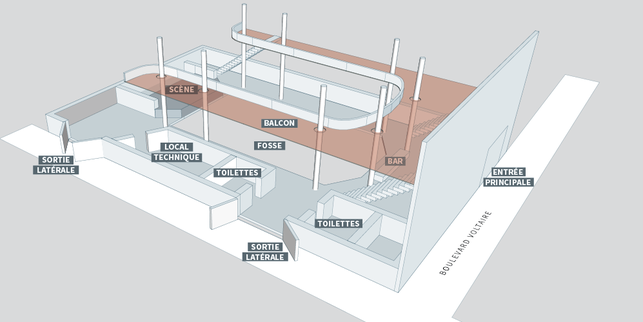


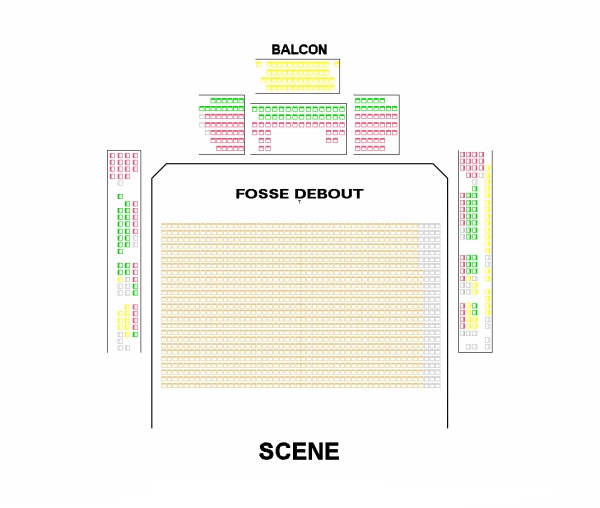


**E3.** « Je vais vous poser quelques questions sur vos émotions et pensées à ce moment-précis ».

**E3.1 Aviez-vous l’impression que votre vie pouvait être en danger ?** (cocher une seule case)

Oui

Non

Je ne sais pas

**E3.2 Aviez-vous l’impression que la vie de vos connaissances** (si applicable) **pouvait être en danger ?** (cocher une seule case)

Oui

Non

Je ne sais pas

Non-applicable (seul dans la salle)

**E3.3 L’évacuation vous semblait-elle possible ?** (cocher une seule case)

Oui

Non

**E4.** « Je vais à présent vous poser quelques questions concernant la suite, **jusqu’à votre sortie du Bataclan.** Pour répondre à ces questions, il est important de garder en tête **qu’il s’agit de vous concentrer sur les pensées, émotions et croyances que vous avez eues à ce moment précis.** Avez-vous vu ou fait ces choses : pas du tout, au moins une fois, plus d’une fois, ou un grand nombre de fois.»

(cocher une seule case par item)

|  | **Pas du tout** | **Au moins une fois** | **Plus d’une fois** | **Un grand nombre de fois** |
| --- | --- | --- | --- | --- |
| **Réactions spontanées** | **0** | **1** | **2** | **3** |
| E3.1.a Avez-vous couru ou cherché à vous enfuir ? |  |  |  |  |
| E3.1.b Avez-vous vu des personnes courir ou chercher à s’enfuir ? |  |  |  |  |
| E3.2.a Avez-vous hurlé ? |  |  |  |  |
| E3.2.b Avez-vous entendu des personnes hurler ? |  |  |  |  |
| E3.3.a Vous-êtes-vous recroquevillé ? |  |  |  |  |
| E3.3.b Avez-vous vu des personnes se recroqueviller ? |  |  |  |  |
| E3.4.a Avez-vous bousculé une personne pour vous protéger ? |  |  |  |  |
| E3.4.b Avez-vous vu des personnes en bousculer d’autres pour se protéger ? |  |  |  |  |
| E3.5.a Êtes-vous resté immobile ? |  |  |  |  |
| E3.5.b Avez-vous vu des personnes rester immobile ? |  |  |  |  |
| E3.6.a Avez-vous essayé d’en savoir plus sur la menace ? |  |  |  |  |
| E3.6.b Avez-vous eu l’impression que les personnes autour de vous essayaient d’en savoir plus sur la menace ? |  |  |  |  |
| E3.7.a Avez-vous cherché à vous agripper à quelqu’un ? |  |  |  |  |
| E3.7.b Les gens cherchaient-ils à s’agripper à une autre personne ? |  |  |  |  |
| **Relations à l’autre** | **0** | **1** | **2** | **3** |
| E3.7.a Avez-vous protégé une personne de votre corps ? |  |  |  |  |
| E3.7.b Avez-vous vu des personnes en protéger d’autres par leur corps ? |  |  |  |  |
| E3.8.a Vous-êtes-vous mutuellement protégé avec quelqu’un ? |  |  |  |  |
| E3.8.b Avez-vous des personnes se protéger mutuellement par leur corps ? |  |  |  |  |
| E3.9.a Avez-vous tenté de rassurer quelqu’un (texto, geste, parole) ? |  |  |  |  |
| E3.9.b Avez-vous vu des personnes tenter d’en rassurer d’autres (texto, geste, parole) ? |  |  |  |  |
| E3.10.a Avez-vous appelé à l’aide (texto, geste, parole) ? |  |  |  |  |
| E3.10.b Avez-vous vu des personnes en appeler d’autres à l’aide (texto, geste, parole) ? |  |  |  |  |
| E3.11.a Avez-vous apporté de l’aide à quelqu’un ? |  |  |  |  |
| E3.11.b Avez-vous vu des personnes apporter de l’aide à une autre ? |  |  |  |  |
| **Communication avec les autres otages** | **0** | **1** | **2** | **3** |
| E3.12.a Avez-vous informé autrui de la nature de la menace (texto, geste, parole) ? |  |  |  |  |
| E3.12.b Avez-vous vu des personnes informer autrui de la nature de la menace  (texto, geste, parole) ? |  |  |  |  |
| E3.13.a Avez-vous informé autrui de la position de la menace (texto, geste, parole) ? |  |  |  |  |
| E3.13.b Avez-vous vu des personnes informer autrui de la position de la menace (texto, geste, parole) ? |  |  |  |  |
| E3.14.a Avez-vous informé autrui de la position des issues de secours (texto, geste, parole) ? |  |  |  |  |
| E3.14.b Avez-vous vu des personnes informer autrui de la position des issues de secours (texto, geste, parole) ? |  |  |  |  |
| E3.15.a Avez-vous informé autrui de votre blessure (si applicable) (texto, geste, parole) ? |  |  |  |  |
| E3.15.b Avez-vous vu des personnes informer autrui de leur blessure (texto, geste, parole) ? |  |  |  |  |
| **Communication avec l’extérieur** | **0** | **1** | **2** | **3** |
| E3.16 Avez-vous tenté de communiquer avec l’extérieur (texto, appel, social media) ? |  |  |  |  |

# E5. « Le dessin ci-dessous représente votre ‘relation à autrui’. En A, vous vous sentiez totalement indépendant d’autrui. En E, vous sentiez que vous-mêmes et autrui ne faisiez qu’un. Pourriez-vous me dire quelle situation correspondait à votre ressenti d’alors (la suite jusqu’à votre sortie du Bataclan) envers (une réponse par item) : »

# Vos proches (famille, amis, connaissances) présents au Bataclan _________________ ?

# Vos proches (famille, amis, connaissances) non-présents au Bataclan _________________ ?

# Le reste de la foule _________________ ?

# Le groupe Eagles of Death Metal _________________ ?

#
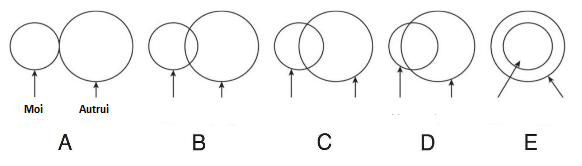


# Partie F.

« Nous avons bientôt fini. Il me reste quelques questions : »

**F1.** Sur une échelle de 0 à 9 (0 représentant « pas du tout » et 9 « totalement »), vous sentiez-vous maître de votre comportement :

# 1) Lorsque vous saviez que quelque chose de grave se passait mais que vous ne saviez pas quoi exactement _________________ ?

# 2) Lorsque vous vous êtes rendus compte qu’il s’agissait d’une attaque à l’arme à feu._________________ ?

# 3) Le reste du temps, jusqu’à la sortie du Bataclan _________________ ?

**F2.** Pourriez-vous indiquer le temps approximatif que vous pensez qu’ont duré les étapes 1 (lorsque vous saviez que quelque chose de grave se passait mais que vous ne saviez pas quoi exactement- indiquer 1 en dessous de la partie correspondante), 2 (lorsque vous vous êtes rendus compte qu’il s’agissait d’une attaque à l’arme à feu) et 3 (le reste du temps, jusqu’à la sortie du Bataclan) ? Indiquez les heures approximatives si vous les connaissez

Etape 1 : _____ sec/min/heure (rayer la mention inutile)
Etape 2 : _____ sec/min/heure (rayer la mention inutile)

Etape 3 : _____ sec/min/heure (rayer la mention inutile)

**F3.** Pourriez-vous me raconter librement ce qui s’est passé quand vous êtes sortis dans la salle (notamment vos relations avec autrui – connaissances ou non) ?

**F4.** Rétrospectivement avez-vous remarqué une certaine entraide pendant l’événement ? Avez-vous remarqué une absence d’entraide ? Si oui, l’entraide était-elle la réaction la plus spontanée (et première) ? Ou est-elle apparue après un certain moment ? Si oui, quand ? Quelles conditions vous ont semblé nécessaires à la mise en place de comportements d’entraide ?

# **MERCI !**

Donner au participant le contact du Dr. Philippe Nuss pour un entretien médical

s’il le souhaite (numéro du secrétariat : 01 49 28 26 56)

# **Date et heure de fin d’entretien : ___________________**
