# Supplementary material for: Nature and determinants of social actions during a mass shooting
Source: PLoS One. 2021 Dec 7;16(12):e0260392. doi: 10.1371/journal.pone.0260392 (PMC8651140; doi:10.1371/journal.pone.0260392)
Supplement: S4 File — (DOCX) [file pone.0260392.s004.docx]

**Nature and determinants of social actions during a mass shooting**

*Short title: Social actions during a mass shooting*

**SUPPORTING INFORMATION**

**S1 Table. Respondents’ demographics.**

| **Sample (N=32; mean age in 2016  = 36.18 y.o., +/- 1.13 SEM)** | **N** | **% of the sample** |
| --- | --- | --- |
| Sex ratio (female/male) | 17 / 15 | 53.1% / 46.9% |
| Level of education* |  |  |
| *BAC* | 6 | 18.75% |
| *BAC+3* | 7 | 21.9% |
| *BAC+5* | 12 | 37.5% |
| *Higher than BAC+5* | 7 | 21.9% |
| Socioprofessional category** |  |  |
| *Artisans, commerçants et chefs d'entreprise* | 2 | 6.25% |
| *Cadres et professions intellectuelles supérieures* | 19 | 59.4% |
| *Professions intermédiaires* | 3 | 9.4% |
| *Employés* | 7 | 21.9% |
| *Ouvriers* | 0 | 0.0% |
| *Etudiants* | 1 | 3.1% |
| With spouse at the Bataclan | 12 | 37.5% |
| With friends at the Bataclan | 23 | 71.8% |
| Hit by a bullet | 9 | 28.1% |
|  |  |  |
|  |  |  |

*Level of education calculation is based on 4 categories: BAC, BAC+3 (with BAC+2 being merged in this category), BAC+5 (with BAC+4 being merged in this category); Higher than BAC+5 for individuals with education after level BAC+5. Note: BAC refers to the French baccalaureate: https://en.wikipedia.org/wiki/Baccalaur%C3%A9at

**Socioprofessional categories are based on French conventions. They roughly translate as follows:

- artisans, commerçants et chefs d’entreprises = craftsmen, shopkeepers, business owners

- cadres et professions intellectuelles supérieures = senior managers and professional occupations

- professions intermédiaires = middle management positions

- employés = employees

- ouvriers = factory workers

- étudiants = students

**Models selections**

The following Supporting .txt files provide full information about each model, namely: value of intercept, regression values for each relevant factor, df (degrees of freedom), LogLikelihood, AICc, delta (delta of AICc), Weight (Akaike Weight calculated out of AICc – using function ‘model.sel’ of package MuMin (v. 1.40.4)).

For all episodes: see uploaded file PONE-D-20-26798_modelsselectiontypology.txt

For episodes where agent is the Respondent: see uploaded file PONE-D-20-26798_modelsselectiontypologypp.txt

**S2 Table. The motivation behind supportive actions.** Motivations are mutually exclusive.

| **Type of motivation** | **Definition** |
| --- | --- |
| INDIVIDUALISTIC | One action in which the welfare of the respondent is the only thing being relevant (and the welfare of others being ignored or deemed irrelevant). |
| MUTUALLY BENEFICIAL (COOPERATION) | One action undertaken by the respondent which benefits both himself/herself and another individual and which is presented as such by the respondent. |
| ALTRUISTIC | One action is undertaken by the respondent which benefits the recipient only and at cost for the respondent, and is presented as such by the respondent. |
