# Supplementary material for: Nature and determinants of social actions during a mass shooting
Source: PLoS One. 2021 Dec 7;16(12):e0260392. doi: 10.1371/journal.pone.0260392 (PMC8651140; doi:10.1371/journal.pone.0260392)
Supplement: Attachment — Submitted filename: PONE-D-20-26798_RespReviewers.docx [file pone.0260392.s007.docx]

**PONE-D-20-26798: Response to Reviewers**

**The Editor**

1. Please ensure that your manuscript meets PLOS ONE's style requirements, including those for file naming.

This has now been done.

2. Please include additional information regarding the interview guide used in the study and ensure that you have provided sufficient details that others could replicate the analyses. For instance, if you developed an interview guide as part of this study and it is not under a copyright more restrictive than CC-BY, please include a copy, in both the original language and English, as Supporting Information.

The original interview file has been uploaded together with the revised manuscript. We believe that a professional translation should be offered before the questionnaire is also used in other scientific works, a translation that we cannot provide.

3. We recommend that authors use the COREQ checklist, or other relevant checklists listed by the Equator Network, such as the SRQR, to ensure complete reporting (<http://journals.plos.org/plosone/s/submission-guidelines#loc-qualitative-research>).

We filled in the checklist, which we provide for your information.

4. Thank you for including your ethics statement: "Permission to conduct the study was received from the Comité de Protection des Personnes (CPP) of the Centre Hospitalier Universitaire Saint-Antoine, Paris, France. All participants provided informed consent.".   a. Please provide additional details regarding participant consent. In the ethics statement in the Methods and online submission information, please ensure that you have specified what type you obtained (for instance, written or verbal, and if verbal, how it was documented and witnessed). If your study included minors, state whether you obtained consent from parents or guardians. If the need for consent was waived by the ethics committee, please include this information.

It has now been clarified that written consent from majors has been obtained, as required by the ethics committee.

b. Once you have amended this/these statement(s) in the Methods section of the manuscript, please add the same text to the “Ethics Statement” field of the submission form (via “Edit Submission”).

This has been added.

5. In your Data Availability statement, you have not specified where the minimal data set underlying the results described in your manuscript can be found. PLOS defines a study's minimal data set as the underlying data used to reach the conclusions drawn in the manuscript and any additional data required to replicate the reported study findings in their entirety. All PLOS journals require that the minimal data set be made fully available. For more information about our data policy, please see <http://journals.plos.org/plosone/s/data-availability>. Upon re-submitting your revised manuscript, please upload your study’s minimal underlying data set as either Supporting Information files or to a stable, public repository and include the relevant URLs, DOIs, or accession numbers within your revised cover letter. For a list of acceptable repositories, please see <http://journals.plos.org/plosone/s/data-availability#loc-recommended-repositories>. Any potentially identifying patient information must be fully anonymized. Important: If there are ethical or legal restrictions to sharing your data publicly, please explain these restrictions in detail. Please see our guidelines for more information on what we consider unacceptable restrictions to publicly sharing data: <http://journals.plos.org/plosone/s/data-availability#loc-unacceptable-data-access-restrictions>. Note that it is not acceptable for the authors to be the sole named individuals responsible for ensuring data access. We will update your Data Availability statement to reflect the information you provide in your cover letter.

The minimal dataset (which here consists of each action reported by Respondents, together with the coded factors) has now been uploaded together with the revised manuscript. It is then now accessible as a Supplementary File. We are grateful if this information can appear as our Data Availability statement. Note that the minimal dataset cannot encompass the full audio tapes because they contain a large number of information that could lead to identification. Given the sensitivity of the topic, we obviously cannot include these.

6. Please expand the acronym “FRM Team” (as indicated in your financial disclosure) so that it states the name of your funders in full. This information should be included in your cover letter; we will change the online submission form on your behalf.

The FRM stands for ‘Fonds pour la Recherche Médicale’. This information now fully appears in the revised manuscript.

7. Please upload a copy of Figure 3, to which you refer in your text on page 18. If the figure is no longer to be included as part of the submission please remove all reference to it within the text.

Figure 3 is now part of Figure 2 (see Figure 2D). We corrected this in the revised manuscript.

8. Please include captions for your Supporting Information files at the end of your manuscript, and update any in-text citations to match accordingly. Please see our Supporting Information guidelines for more information: http://journals.plos.org/plosone/s/supporting-information

This has been done.

**Reviewer #1**

This paper describes an extremely interesting and useful study. The study is based on an impressive dataset. Data collection and analysis were conducted appropriately. The interpretation of results follows logically from the data. The writing is clear. The study is contextualized through an up-to-date literature review.
However, I have some suggestions for the authors to consider to sharpen the paper. Most of these have to do with justification and communication, as well as some decision-making.

We are extremely grateful Reviewer #1 found our manuscript interesting and enjoy reading it. We thank him for his excellent comments that have made our manuscript much stronger.

Introduction and rationale
The stated rationale – the role of distance to safety and danger – seems to me less important than another reason for this study. There are two points here. First, the question of the relation of support behaviours to danger and possibility of escape has indeed been addressed previously. It is a hypothesis of classical panic theories (which predicts that people don’t help when they face danger, they become apathetic when they can’t escape). The relation between helping and threat/damage has also been addressed a little in the more recent social identity studies:
Drury et al. (2009) International Journal of Mass Emergencies & Disasters;
Drury et al. (2009). Behavior Research Methods
Bartolucci, A., Casareale, C., & Drury, J. (2021). Cooperative and competitive behaviour among passengers during the Costa Concordia disaster. Safety Science, 134. <https://doi.org/10.1016/j.ssci.2020.105055>
Ntontis, E., Drury, J., Amlôt, R., Rubin, G. R., Williams, R., & Saavedra, P. (2020). Collective resilience in the disaster recovery period: Emergent social identity and observed social support are associated with collective efficacy, wellbeing, and the provision of social support. British Journal of Social Psychology <https://doi.org/10.1111/bjso.12434>
The authors should be a little more specific about what they can add to this existing literature.

Second, the main interest for me and many others will be the fact that this is a detailed study of public behaviour in response to a marauding terrorist attack. This is important and interesting because prima facie we might perhaps expect differences in how people respond in this situation compared to other kinds of emergencies (e.g., earthquakes, fires) and indeed other kinds of terrorist attacks (e.g. bombings).
I am aware, of course, that Plos One doesn’t consider novelty/ rationale to be relevant, but for completeness I think the authors should consider this point.

We have now enriched the presentation of, what actually was the original rationale of our study, i.e., the role of distance to safety and danger on supportive behavior in emergency circumstances. We notably included the important references provided by Reviewer #1.

We also mention the main interest for the Reviewer #1 (and undeniably, the rest of the research community), i.e., that this study is about a mass shooting.

Thank you!

The concept of ‘fusion’ needs references and justification. In the literature, the concept is identity fusion. The authors refer to ‘fusion’ and ‘emotional fusion’ which looks ad hoc. In the literature ‘fusion’ occurs for those have a very strong identification with. Are the authors therefore referring to emergent identification (as in the social identity model of emergent groups) or pre-existing strong connections between people, or both?

Thanks for this very important comment. We used the ‘inclusion of others in the self’ scale, which we now refer to, and change our construct for ‘interpersonal closeness’. In the Introduction, we note that this scale was chosen to measure how close people felt with other crowd members, as this could explain commitment to act with or in support with others. The Discussion offers a note on the theoretical consequences of the choice of this scale, which we believe should be improved by future studies.

Method
The references to June 2015 are confusing. This is before the attack. Do the authors mean 2016?

We indeed meant 2016 and we are grateful this mistake has been spotted by Reviewer #1.

Lines 171 on: ‘Respondents were also asked about their feeling of fusion to familiar others at the Bataclan, familiar others not present at the Bataclan, the rest of the crowd and the music band, using a scale ranging from A (no fusion with others) to E (complete fusion with others).’
What was the word used in French? Why ‘fusion’? Where did the measure come from? If the scale was developed by someone else, it needs a reference. If not, is it based on something we can have confidence in?

We now include a reference to the scale we used (inclusion of others in the self). The interview guide is also part of the resubmission files.

Supporting information should include the verbatim interview schedule.

As requested by both the Editor and Reviewer #1, the ‘interview guide’ has been included.

p. 14 The definition of ‘episode’ is circular (it contains the word ‘episode’) and should be tightened. Social action?

We clarified this definition.

p. 15 ‘Each of the social episodes was then categorized by GD following a built-in typology (see Supporting Information for full details).’
I would prefer to see this in the main document.

This is now also present in the main document.

I would also recommend presenting an illustrative quotation for each category too. For an interview study, we should hear some of the voices of the participants.

This has been done (see Table 1).

p. 15 ‘Finally, SOCIAL NORMS was used when there was clear evidence of people setting up a stable activity which requires the instauration of a social norm, which was explicitly mentioned by the Respondent.’
This is unclear and underlines my point that examples (quotations from interviewees) should be provided.

We now clarify this definition, by using a quotation from one respondent (see Table 1).

p. 15 ‘Agreement between GD, JRM and JG and each of the three external judges to categorize the social episodes into the proposed categories was substantial (all ks > .6).’
So less than 7? It’s low. Can it be improved?

We now include all values, which were k with judge 1 = 0.78; k with judge 2 = 0.63, and k with judge 3 = 0.77. k = 0.63 was the lowest value found, with the other two suggesting substantial agreement. We would be happy to include the confusion matrices as part of the Supplementary Information if relevant, although we are unsure how they could be telling. Some confusion arose between ‘altruistic’ and ‘cooperative’, and some between ‘cooperative’ and ‘individualistic’.

p. 17 ‘As a measure of SOCIAL SUPPORT, we took, for each Respondent, the absolute number of familiar people they came with at the concert’
The label is confusing. It is an indication of existing relationship, not social support given or received. I suggest changing it to a different label.

We understand the confusion and we now decided to use the term ‘FRIENDS’.

Results
How many reported ‘stampedes’? How many ‘stampedes’ do the authors estimate took place?

It is unfortunately impossible to answer convincingly to this question. We now avoid this term, for it suggests people are willingly trampling others. As the Reviewer knows, this type of collective behavior can happen by the sole law of physics (a lack of space create opportunities for so-called ‘stampedes’).

P. 22 ‘They were also more likely to be supportive as participants felt emotionally fused with others’
Wording could be amended to:
They were also more likely to be supportive the more participants felt emotionally fused with others

This has been changed. Thank you!

Discussion
p. 28 ‘As a matter of fact, supportive actions were more frequently attributed to others’
As noted in other studies (Drury, 2009, London bombings)

This reference has been added.

p. 25 ‘exposure to danger fosters supportive norms through perceived common fate and associated shared social identity’
A little imprecise as an account of the SI approach to emergencies, which focuses on collective self-interest rather than norms (see Drury, 2018).

Thank you – we have corrected this.

Figures
Figures C and D have typos.

These have been fixed. Thank you for your excellent comments!

**Reviewer #2**

This paper presents interesting insights into people’s reactive behaviours, and the underlying motives of those behaviours, during a life-threatening situation. If my understanding is correct, the authors build upon previous to explore how physical constraints are associated with the prevalence of supportive and unsupportive behaviours, and code responses in more detail to understand the psychological motivations (cooperative, altruistic, individualistic or self-serving) of their actions. I believe the in-depth examination of survivor accounts of the Bataclan attacks has the potential to be a valuable contribution to the literature. However, I have some reservations and questions, specifically with regards to details surrounding HOW the present work extends previous research on the topic, and the rationale for aspects of the coding scheme, analyses etc. I outline these below in no particular order.

We would like to thank Reviewer #2 for her/his appreciation of our manuscript, a diligent reading, and excellent comments. These have all been taken into account to prepare the revised version.


1. First, I think elaboration for some of the terminology used in the Abstract (e.g., “supportive behaviours”, “individualistic”) would be helpful, especially considering the general readership of this journal.

We now elaborate on each of these terms in the Abstract.

2. Elaboration on the tenets of the three accounts developed to understand reactive behaviours in dangerous contexts – and the supporting empirical evidence - is also needed. Relatedly, previous research on this topic should be described in more detail. In the present format, it is not clear what evidence led the authors to decide to not only code the nature of social behaviour, but comments about physical constraints and motives. Having this clear distinction between the past and present research would emphasise the importance and contribution of the paper.

This has been elaborated too. We are grateful to the Reviewer #2 but also Reviewer #1 for their call to do so.

3. I understand the authors adopted a bottom-up/exploratory approach but I was left wondering about potential hypotheses (again, possibly informed by past work or the relevant theories). Further, the aims outlined in the Introduction do not clearly map onto the coding and analyses sections. I suggest some more elaboration and restructuring here.

We restructured the Introduction to enhance the liaison with the rest of the manuscript.

4. Has the construct of “fusion” appeared in previous literature? Or could it relate to other psychological constructs (e.g., empathic concern)? It seems to be an important psychological predictor in the current report, and I’d be interested in learning more about the rationale for including this measure.

As also requested by Reviewer #1 too, we now offer more details on this concept, with a reference to the scale being used.

5. It would be helpful to include the rationale for focusing on the three specific time periods of the attack during the interview.

We now explain that this was the optimal way to test whether supportive behavior emerge early on or not. We now explain this, and re-elaborate on why this was not further pursued in the analysis.

6. I empathise with the difficulty of coding complex open-ended responses but the inter-rater reliability for the coding of participant motives are lower than the accepted standard (all ks > .6) – is there a reason why this might be the case?

As we also told Reviewer #1: We now include all values, which were: k with judge 1 = 0.78; k with judge 2 = 0.63, and k with judge 3 = 0.77. So k = 0.63 was the lowest value found, with the other two suggesting substantial agreement. We would be happy to include the confusion matrices as part of the Supplementary Information if relevant, although we are unsure how they could be telling. Some confusion arose between ‘altruistic’ and ‘cooperative’, and some between ‘cooperative’ and ‘individualistic’.

7. Why was the median score used for the estimation of participants’ feelings of fusion?

This was done to obtain one unique value across the episodes narrated by Respondent. We now explain this.

8. The chosen models seem appropriate but, related to point 3., it is sometimes hard to follow the rationale for entering the predictors of interest.

We now hope that this is clearer, notably thanks to our response to point 3.

9. What does the x-axis represent in Figure 2 D. (motivation for supportive actions)?

These are each participant who reported a supportive action as agent. This has now been clarified.

10. Is there a possibility that unsupportive actions, and antisocial motives, are underreported here?

This is possible indeed, and we now explain it in the Discussion.

11. There is a typo on page 20: “In a previous study, it has been shown that part of the pre-evacuation responses to perceived danger in survivors of the WTC attacks in 2011 involved informing relatives, sometimes via phone calls” This should refer to the World Trade Center attacks in 2001.

We now correct this. Apologies for this typo.
